# Human Umbilical Vein Endothelial Cells foster conversion of CD4^+^CD25^−^Foxp3^−^ T cells into CD4^+^Foxp3^+^ Regulatory T Cells via Transforming Growth Factor-β

**DOI:** 10.1038/srep23278

**Published:** 2016-03-18

**Authors:** Anika Oettel, Mario Lorenz, Verena Stangl, Serban-Dan Costa, Ana Claudia Zenclussen, Anne Schumacher

**Affiliations:** 1 Experimental Obstetrics and Gynecology, Medical Faculty, Otto-von-Guericke University, Gerhart-Hauptmann-Straße 35, 39108 Magdeburg, Germany; 2 Medical Clinic for Cardiology and Angiology, Campus Mitte, Charite – Universitätsmedizin Berlin, Germany, Charitéplatz 1, 10117 Berlin, Germany; 3 DZHK (German Center for Cardiovascular Research), partner site Berlin, Oudenarder Straße 16, 13347 Berlin, Germany; 4 University Women’s Clinic, Otto-von-Guericke University, Gerhart-Hauptmann-Straße 35, 39108 Magdeburg, Germany

## Abstract

Trans-placental cell trafficking is a naturally occurring process during pregnancy that results in the direct recognition of foreign maternal antigens by fetal tissue and vice versa. Immigration of potentially harmful allo-reactive maternal T cells into fetal circulation may provoke anti-fetal immune responses. However, the contact with fetal tissue may favor differentiation of maternal immune cells into cells with a regulatory phenotype. Human Umbilical Vein Endothelial Cells (HUVECs) possess immune-regulating properties and are one of the first fetal cells to get in contact with foreign maternal immune cells. Therefore, here we studied whether HUVECs induce the conversion of maternal T cells into regulatory T (Treg) cells. Moreover, we assessed whether this response is changing according to the sex of the HUVECs. Both female and male HUVECs induced the conversion of maternal T cells into Treg cells which is partially mediated via TGF-β. Female HUVECs showed a stronger capacity to induce Treg cells compared to male HUVECs. Our findings propose that HUVECs contribute to fetal-maternal tolerance by the increase of the Treg cell population. Sex-specific differences in Treg cell induction may partly account for the disparities on the incidence of infectious and autoimmune diseases between both sexes during early childhood.

Trans-placental bi-directional cell transfer between mother and fetus is a naturally occurring process during pregnancy and results in fetal and maternal microchimerism^1^,^2^,^3^. Consequently, during and after pregnancy the maternal immune system is challenged by the presence of foreign fetal antigens while the fetal immune system is challenged by the presence of foreign non-inherited maternal antigens. Being a chimera has beneficial and detrimental consequences for both individuals.

Maternal microchimerism (MMC) has been proven for various immune cell populations in tissues from fetuses, neonates and adult progenies in almost all organs^4^,^5^,^6^. For fetuses and neonates, this may result in even more pronounced consequences than for adults, as their immune system differs from the mature immune system. Interestingly, autoimmune disorders that primarily affect newborns and children have been associated with MMC^7^,^8^,^9^,^10^,^11^ suggesting long-lasting effects in the offspring. During pregnancy, it can be hypothesized that some of the immigrated maternal immune cells are allo-reactive, hence promote anti-fetal immune responses and have the potential of provoking fetal rejection. The contact with fetal antigens, however, may promote the differentiation of potential harmful maternal immune cells into fetus-friendly cells with a suppressive phenotype such as regulatory T (Treg) cells.

Human Umbilical Vein Endothelial Cells (HUVECs) lining the wall of umbilical veins are very probably the first fetal cells getting in contact with immigrated maternal cells. This includes monocytes, natural killer cells, dendritic cells, T and B cells (reviewed in^12^,^13^) all detectable in cord blood. Interestingly, the frequency of maternal cells compared to total cells in cord blood has been shown as high as 1%^14^. In a detailed analysis of the cellular composition of maternal neonatal cord blood, Mold and colleagues revealed a predominance of maternal hematopoietic cells. Besides maternal CD14^+^ monocytes and CD19^+^ B cells the authors detected maternal CD3^+^ T cells including both CD8^+^ cytotoxic T cells and CD4^+^ T helper cells^15^. Moreover, several studies suggest the immune-modulating properties of HUVECs including the recruitment, activation and suppression of immune cell populations^16^,^17^,^18^.

HUVECs are not only in close contact to immigrated maternal immune cells that present foreign antigens, but are also capable of regulating them. We therefore studied whether HUVECs can influence T cell proliferation and induce Treg cells from CD4^+^ T cells. Treg cells represent a unique T cell subpopulation with key functions in fetal tolerance establishment and maintenance (reviewed in^19^). We further sought to elucidate whether HUVECs from female or male donors have different abilities in modulating the maternal immune system.

## Results

### HUVECs did not influence the proliferation of CD4^+^ T cells

**Figure 1:**
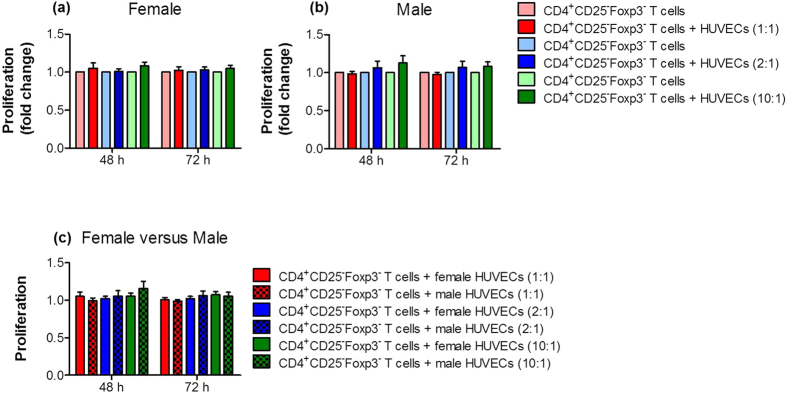
HUVECs did not alter proliferation of CD4^+^ T cells. CD4^+^CD25^−^Foxp3^−^ T cells were co-cultured with primary female or male HUVECs at cell-to-cell ratios of 1:1; 2:1 and 10:1 (T cells:HUVECs). After 48 and 72 hours, proliferation of CD4^+^CD25^−^Foxp3^−^ T cells was determined. Neither female **(a)** nor male **(b)** HUVECs altered T cell proliferation. Direct comparisons between female and male HUVECs **(c)** revealed no differences. Co-cultures with HUVECs are given as fold change of controls (T cells cultured alone). Samples from 16 normal pregnant women were analyzed in duplicates. Data are presented as means ± S.E.M. Statistical analysis among groups was performed using Two-way-ANOVA followed by Bonferroni correction for multiple comparisons.

First, we studied whether primary female or male HUVECs affect the proliferation of CD4^+^ T cells obtained from healthy pregnant women. We co-cultured HUVECs with CD4^+^CD25^−^Foxp3^−^ T cells at different cell-to-cell ratios for 48 and 72 hours. Neither female nor male HUVECs altered the proliferation of CD4^+^CD25^−^Foxp3^−^ T cells, irrespective of the cell-to-cell ratio and the time point analyzed (Fig. 1a,b). Further, a direct comparison between female and male HUVEC co-cultures revealed no difference (Fig. 1c).

### HUVECs induced the conversion of CD4^+^ T cells into Treg cells

**Figure 2:**
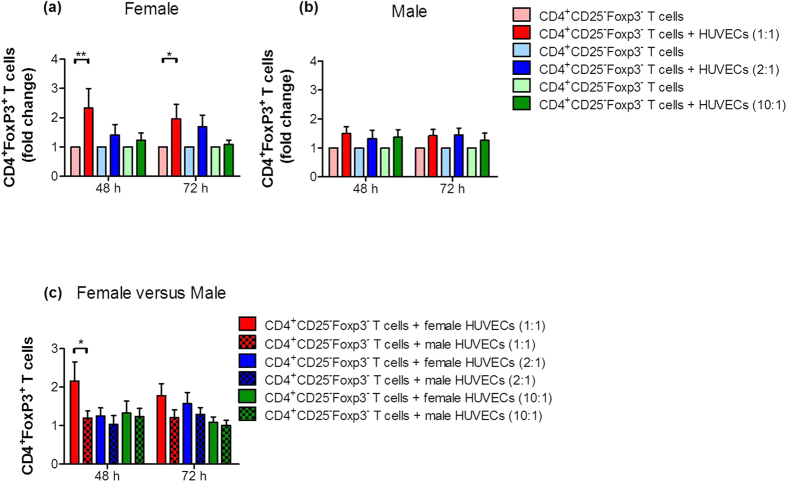
HUVECs induced Treg cell differentiation from CD4^+^ T cells. CD4^+^CD25^−^Foxp3^−^ T cells were co-cultured with primary female or male HUVECs at cell-to-cell ratios of 1:1; 2:1 and 10:1 (T cells:HUVECs). After 48 and 72 hours, the number of CD4^+^Foxp3^+^ T cells was analyzed. HUVECs of both sexes induced Treg cell generation. However, Treg cell number was significantly increased only in co-cultures with female HUVECs **(a)** at a cell-to-cell ratio of 1:1 at both time points, while male HUVECs **(b)** stimulated only a slight increase. Results are presented as fold change of controls (T cells cultured alone). Comparison between both sexes revealed a significant stronger potential of female HUVECs to induce Treg cells compared to male cells **(c)**. Samples from 16 normal pregnant women were analyzed in duplicates. Data are presented as means ± S.E.M. Statistical analysis among groups was performed using Two-way-ANOVA followed by Bonferroni correction for multiple comparisons. *p < 0.05; **p < 0.01.

Next, we analyzed the ability of primary female or male HUVECs to induce the conversion of CD4^+^ T cells into Treg cells. In line with the literature, Treg cells were identified as CD4^+^Foxp3^+^ cells^20^. HUVECs were co-cultured with CD4^+^CD25^−^Foxp3^−^ T cells at different cell-to-cell ratios and the number of CD4^+^Foxp3^+^ T cells was determined after 48 and 72 hours. We observed an increase in the number of CD4^+^Foxp3^+^ T cells during co-culture of CD4^+^ T cells with HUVECs of either sex at both time points (Fig. 2a,b). However, the increase in the number of Treg cells was statistically significant only in co-cultures with female HUVECs at a cell-to-cell ratio of 1:1 (Fig. 2a) but not in co-cultures with male HUVECs at any cell-to-cell ratio (Fig. 2b). After comparing female with male HUVECs, female HUVECs had a significant stronger potential to induce Treg cells compared to male HUVECs in a cell ratio of 1:1 after 48 hours (Fig. 2c). Representative flow cytometry plots of the co-cultures showing real percentages are provided in Supplementary Figs S1 and S2.

### HUVECs did not attract CD4^+^ T cells but provoke their conversion into Treg cells through soluble factors

**Figure 3:**
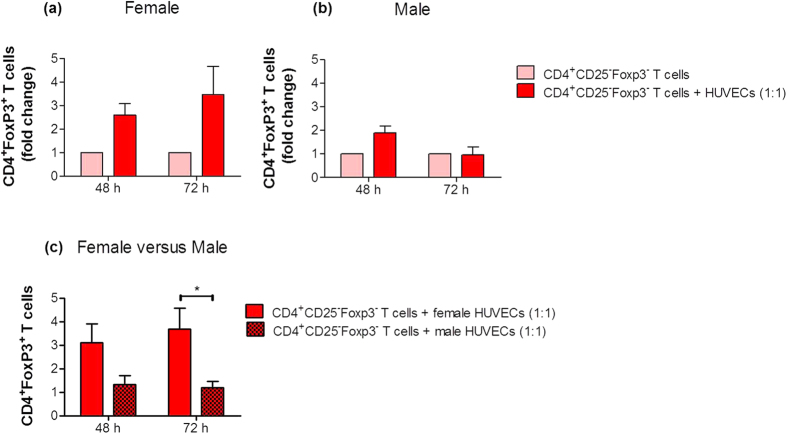
Treg cell induction by HUVECs was mediated through secretion of soluble factors. CD4^+^CD25^−^Foxp3^−^ T cells were co-cultured with primary female or male HUVECs at a cell-to-cell ratio of 1:1 (T cells:HUVECs) in a trans-well system. After 48 and 72 hours, the number of CD4^+^Foxp3^+^ T cells was analyzed. Female **(a)** and male **(b)** HUVECs stimulated Treg cell generation to a different extent. Co-cultures with HUVECs are presented as fold change of controls (T cells cultured alone). Female HUVECs showed a significant higher capacity to induce Treg cells **(c).** Samples from three normal pregnant women were analyzed in duplicates. Data are presented as means ± S.E.M. Statistical analysis among groups was performed using Two-way-ANOVA followed by Bonferroni correction for multiple comparisons. *p < 0.05.

To elucidate whether HUVEC-induced Treg cell generation from CD4^+^ T cells depends on direct cell-to-cell contacts or is mediated through soluble factors secreted by HUVECs, we next determined the ability of female or male HUVECs to attract CD4^+^ T cells. We analyzed the number of migrated CD4^+^CD25^−^Foxp3^−^ T cells to female or male HUVECs after 4, 8, 24 and 48 hours. Neither female nor male HUVECs favored the migration of CD4^+^CD25^−^Foxp3^−^ T cells at any time point (see Supplementary Fig. S3). Next, we assessed the conversion of CD4^+^CD25^−^Foxp3^−^ T cells into CD4^+^Foxp3^+^ Treg cells by soluble factors by preventing direct cell-to-cell interaction between HUVECs and CD4^+^ T cells in a trans-well system at a 1:1 cell-to-cell ratio. We observed an increased conversion into CD4^+^Foxp3^+^ Treg cells in the presence of female HUVECs for 48 and 72 hours (Fig. 3a). The presence of male HUVECs resulted in a slight and not statistically significant augmentation of CD4^+^Foxp3^+^ Treg cells after 48 hours (Fig. 3b). The comparison between female and male HUVECs confirmed our previous observations. Female HUVECs possess an increased potential to induce Treg cells from CD4^+^ T cells compared to male HUVECs (Fig. 3c).

### HUVECs secreted high amounts of TGF-β but not IL-10

Since HUVECs failed to stimulate migration of CD4^+^CD25^−^Foxp3^−^ T cells but could induce Treg cell generation through the secretion of soluble factors, we focused our investigations on two cytokines, IL-10 and TGF-β, known for their participation in Treg cell induction^21^,^22^. However, we could not detect IL-10 secretion by unstimulated female or male HUVECs after 48 and 72 hours (data not shown). In contrast, we found high levels of TGF-β in the supernatants of primary female and male HUVECs that have been cultured for 48 (female: 721.7 ± 46.35 pg/ml; male: 766.6 ± 37.14 pg/ml) and for 72 hours (female: 905.1 ± 40.33 pg/ml; male: 822.9 ± 41.45 pg/ml).

### Blockage of TGF-β impaired HUVEC-induced Treg generation

**Figure 4:**
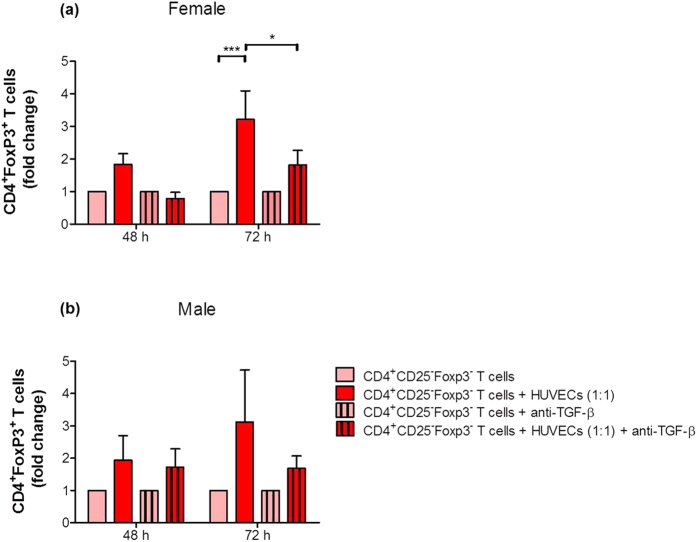
Blockage of TGF-β impaired HUVEC-mediated Treg cell differentiation. CD4^+^CD25^−^Foxp3^−^ T cells were co-cultured with primary female or male HUVECs at a cell-to-cell ratio of 1:1 (T cells:HUVECs). TGF-β was blocked in some experiments. After 48 and 72 hours, the number of CD4^+^Foxp3^+^ T cells was determined. Female HUVECs **(a)** significantly increased the conversion to Treg cells after 72 hours which was significantly suppressed by a neutralizing TGF-β antibody. Similar results, although not statistically significant, were obtained with male HUVECs **(b).** Co-cultures with HUVECs are given as fold change of controls (T cells cultured alone). Samples from four normal pregnant women were analyzed in duplicates. Data are presented as means plus S.E.M. Statistical analysis among groups was performed using Two-way-ANOVA followed by Bonferroni correction for multiple comparisons. *p < 0.05; ***p < 0.001.

To study whether TGF-β is involved in HUVEC-mediated conversion of CD4^+^ T cells into Treg cells, we performed blocking experiments. We focused our analysis on the 1:1 cell-to-cell ratio between HUVECs and CD4^+^CD25^−^Foxp3^−^ T cells. In some approaches we blocked TGF-β by using a monoclonal antibody directed against TGF-β1, TGF-β2 and TGF-β3. Blocking of TGF-β impaired the HUVEC-induced conversion of CD4^+^CD25^−^Foxp3^−^ T cells into CD4^+^Foxp3^+^ Treg cells in both sexes (Fig. 4a,b). However, the blocking effect was more pronounced after co-cultures with female HUVECs and reached statistical significance after 72 hours (Fig. 4a,b).

## Discussion

In the present study we identified HUVECs as inducers of Treg cells from maternal CD4^+^ T cells, at least in part through a TGF-β dependent pathway. Moreover, we observed sex-specific differences in Treg cell induction. We found primary female HUVECs to be more potent than primary male HUVECs in directing the maternal immune system.

For long time it was thought that mother and fetus are completely separated by the placenta and rejection of the fetus is prevented in normal progressing pregnancy because of a missing recognition of the foreign fetal antigens or in other words immune ignorance. However, nowadays there is scientific consent that both fetal and maternal cells trespass the placenta, leading to the phenomenon of fetal and maternal microchimerism with beneficial and detrimental consequences for both the mother and the offspring, which seems to depend on the magnitude of the trespassing. During pregnancy, tolerance towards the foreign fetal and maternal antigens is achieved by an active immune regulation. Most studies concentrate on the modulation of anti-fetal immune responses in the decidua, the maternal part of the fetal-maternal interface. However, it can be speculated that some maternal immune cells immigrating into the fetal circulation possess anti-fetal immune reactivity and may initiate processes that can harm the fetal tissue. Thus, we hypothesized that regulation of maternal immune cells may not only takes place on the maternal but also on the fetal side of the placenta. To test this hypothesis, we studied the potential of female and male HUVECs to contribute to fetal tolerance by altering the proliferation of maternal CD4^+^ T cells and/or induce Treg cells. We found no influence of HUVECs on the proliferation of CD4^+^ T cells. This is in line with observations from Adams and colleagues who demonstrated that “resting” (non-activated) allogeneic HUVECs stimulate IL-2 production, but not proliferation of CD3^+^ PBMCs. Interestingly, in the presence of submitogenic IL-2 concentrations, HUVECs induced strong T cell proliferation^23^. In our *in vitro* culture system, primary HUVECs did not produce IL-2 (data not shown) and exogenous IL-2 was not added to the co-cultures. This may explain why HUVECs were not able to induce T cell proliferation in our study.

Despite the lack of an effect on proliferation, both female and male HUVECs induced Treg cells from maternal CD4^+^ T cells. As Treg cells are key players in the suppression of allo-reactive immune responses^24^ and are critically involved in fetal tolerance (reviewed in^19^), we suggest that Treg induction by HUVECs may represent one of the pathways through which HUVECs contribute to fetal acceptance. Several studies confirmed the presence of Treg cells in cord blood and analyzed their phenotype and functionality^25^,^26^,^27^,^28^,^29^. While Mold and colleagues proved the existence of maternal CD4^+^ T cells in neonatal cord blood^15^, none of the other studies provided evidence about the origin of the cord blood derived Treg cells. Those Treg cells may be of fetal or maternal origin or represent a pool of both. This question should be addressed in a future study, where Treg cells can be sorted from cord blood of male fetuses followed by a XX karyotype analysis for the detection of maternal cells.

After confirmation of a HUVEC-mediated Treg induction, we wondered whether this effect relies on a direct cell-to-cell interaction or is mediated by soluble factors secreted by HUVECs. Primary HUVECs did not attract maternal CD4^+^ T cells that would facilitate conversion into Treg cells by direct cell-to-cell contact. Additionally, HUVECs induced Treg cells also when spatially separated from CD4^+^ T cells. This strongly suggests that HUVECs can exert their effect on CD4^+^ T cells by secreting a soluble factor that favors Treg cell conversion. We subsequently identified TGF-β, a potent Treg cell inducer from CD4^+^ CD25^−^ precursors^30^, as a potential candidate for HUVEC-mediated Treg cell generation. Previous *in vitro* studies in the human and murine system demonstrated that TGF-β induces Foxp3 gene expression in CD4^+^CD25^−^ naïve T cells. Foxp3 induction was further associated with a transition into T cells with a regulatory phenotype and a potent immunosuppressive potential^22^,^31^. *In vivo*, TGF-β promoted the local expansion of the CD4^+^CD25^+^ Treg cell pool and restricted the progression of autoimmunity^32^,^33^.

In our study, both female and male HUVECs secreted high levels of TGF-β and its blockage in co-cultures of HUVECs and CD4^+^ T cells impaired Treg cell induction. Thus, our results suggest a pathway mediated at least partially by TGF-β for generation of Treg cells by HUVECs. In line, Carambia and colleagues showed that Treg cell induction by liver sinusoidal endothelial cells is TGF-β dependent^34^. IL-10 was not involved in the HUVEC-induced positive effect on Treg cell generation. As TGF-β blockage could not completely abrogate the HUVEC-mediated Treg cell generation, we cannot exclude that other soluble factors or direct cell-to-cell interactions may also be involved. Moreover, fetal specific antigens may influence the interaction between HUVECs and T cells and thereby the number of converted Treg cells. Our experiments showed that CD4^+^ T cells from pregnant but not from non-pregnant women are susceptible for a HUVEC effect. However, it remains to be elucidated which are the pregnancy-associated factors that influence T cell susceptibility towards HUVECs.

We were also interested to understand whether there are sex-specific differences in the capacity of primary female and male HUVECs to promote Treg cell differentiation. We observed that female HUVECs had a stronger potential to induce Treg cells than male HUVECs. This finding might be attributed to differences in immune-related parameters between male and female cells. Indeed, in our previous study we found sex-specific differences in the expression of immune-relevant genes between male and female HUVECs. Altogether, more than 70 immune-related genes were higher expressed in female HUVECs^35^. Among these genes were members of the complement system, interleukin receptors, MHC molecules, the toll-like-receptor 10 and the co-stimulatory molecule CD86. We are currently investigating secreted proteins in cell culture supernatants from unstimulated female and male HUVECs. So far, we could identify a higher secretion of MCP-1 and IL-8 in male HUVECs as compared to female HUVECs. Although all of the above differentially expressed molecules are potential candidates for the observed sex-specific effects, a direct influence on Treg cell induction has not been reported in the literature so far. Thus, functional consequences of the observed transcriptional differences between male and female on Treg cell induction remain to be elucidated. Differential numbers of Treg cells generated in female and male fetuses may provide explanations for sex-specific variations in the susceptibility to autoimmune and infectious diseases that can be observed during childhood^36^.

**Figure 5:**
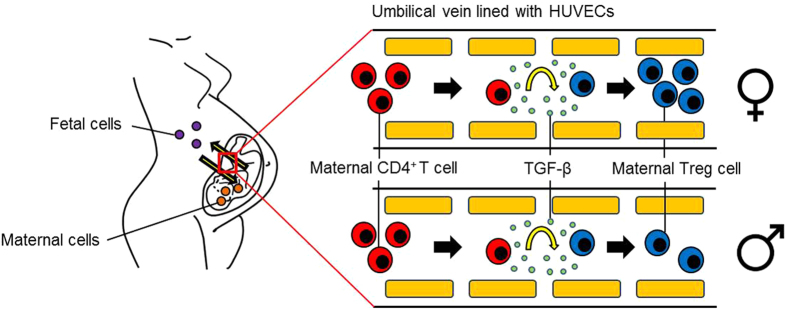
Hypothetical scenario of the HUVEC-mediated Treg cell induction. During pregnancy, potential harmful maternal T cells are able to trespass the placenta. On the fetal side, the immigrated maternal T cells get in contact with HUVECs which are capable to induce their conversion into pregnancy-protective Treg cells. Female HUVECs possess a stronger potential to induce Treg cell conversion than male HUVECs.

In conclusion, we propose that maternal T cells trespass the placenta and get in contact with HUVECs. During pregnancy, HUVECs may prevent anti-fetal immune responses by converting allo-reactive T cells into pregnancy-protective Treg cells, and this effect is apparently sex-specific (Fig. 5).

## Methods

### Ethical approval

Isolation of primary HUVECs from umbilical cords of normal pregnant women after birth was performed at the Department of Cardiology and Angiology (CCM), Charité-Universitätsmedizin Berlin, Germany. The procedure was carried out in accordance to local university guidelines and with the principles outlined in the Declaration of Helsinki. Peripheral blood samples were obtained from normal pregnant women in their third trimester (n = 25; age: 29.1 ± 4.64; week of gestation: 31.9 ± 4.08). Sampling was conducted by the clinicians of the University Women’s Clinic in Magdeburg, Germany. All experimental protocols were approved by the Ethics Board at the University of Magdeburg (study 28/08). All pregnant women gave their written consent after they were informed in detail about the aim of the study.

### Isolation and culture of HUVECs

Primary human umbilical vein endothelial cells (HUVECs) were isolated as described^44^. In brief, umbilical veins were rinsed twice with Hank’s buffered salt solution (HBSS), and after removing of HBSS, one end of the cords was sealed and the umbilical veins were filled with 10 ml of collagenase type II (1 U/ml, Biochrom KG, Berlin, Germany). The digestion was done at 37 °C for 15 minutes in the incubator. Detached cells were then released by flushing the veins with HBSS. The mixture was centrifuged at 1200 rpm for 5 minutes and the cell pellets were resuspended in medium M199. The purity of the HUVEC population following this isolation protocol was >95%^35^. Cells were cultured in medium M199 (Gibco BRL, Carlsbad, CA, USA) supplemented with 20% FCS, 12 μg/ml ECGS (endothelial cell growth supplement, PromoCell, Heidelberg, Germany), 5 U/ml heparin, 2 mM L-glutamine, 5 μg/ml ascorbic acid, 5 μg/ml glutathione, 100 U/ml penicillin, and 100 μg/ml streptomycin. The medium was replaced every 2–3 days. Cells were sub-cultured after treatment with 0.01% trypsin/EDTA. For each experiment HUVECs from different female and male donors were used in passage 5.

### Isolation of CD4^+^CD25^−^Foxp3^−^ T cells

Peripheral blood mononuclear cells (PBMCs) were obtained by Ficoll-Paque^TM^ Plus (GE Healthcare, Uppsala, Sweden) density gradient centrifugation. Afterwards CD4^+^CD25^−^ T cells were isolated by magnetic-activated cell sorting (MACS) using the CD4^+^CD25^+^ Regulatory T cell Isolation Kit (human) from Miltenyi Biotec, Bergisch Gladbach, Germany. All steps were performed under sterile conditions following the instructions of the manufacturer. After isolation, cells were analyzed for CD4, CD25 and Foxp3 expression by flow cytometry. Our analysis revealed a purity of >98% of CD4^+^CD25^−^Foxp3^−^ T cells.

### Proliferation assay

To determine proliferation of CD4^+^CD25^−^Foxp3^−^ T cells, they were stained with carboxyfluorescein diacetate succinimidyl ester (CFDA-SE; Invitrogen, Karlsruhe, Germany) directly after MACS isolation. Then, CD4^+^CD25^−^Foxp3^−^ T cells were co-cultured with female and male HUVECs in different cell-to-cell ratios for 48 and 72 hours. 1 × 10^4^ HUVECs were cultured with either 1 × 10^4^ (ratio 1:1), 2 × 10^4^ (ratio 1:2), or 1 × 10^5^ (ratio 1:10) CD4^+^CD25^−^Foxp3^−^ T cells in HUVEC complete medium. Proliferation was assessed by measuring CFSE intensities with flow cytometry^37^.

### Conversion assays

In a first set of experiments, CD4^+^CD25^−^Foxp3^−^ T cells were co-cultured with primary female or male HUVECs at different cell-to-cell ratios for 48 and 72 hours. 1 × 10^4^ HUVECs were cultured with either 1 × 10^4^ (ratio 1:1), 2 × 10^4^ (ratio 1:2) or 1 × 10^5^ (ratio 1:10) CD4^+^CD25^−^Foxp3^−^ T cells.

In a second set of experiments, 5 × 10^4^ CD4^+^CD25^−^Foxp3^−^ T cells were co-cultured with 5 × 10^4^ primary female or male HUVECs at a 1:1 cell-to-cell ratio for 48 and 72 hours. HUVECs (lower chamber) were separated from CD4^+^CD25^−^Foxp3^−^ T cells (upper chamber) by a semi-permeable membrane (1 μm pore size) using a two-chamber trans-well system. The pore size of the membrane prevents direct intercellular interactions between HUVECs and T cells. Both cell types were cultured in HUVEC complete medium.

In a third set of experiments, 1 × 10^4^ CD4^+^CD25^−^Foxp3^−^ T cells were co-cultured with 1 × 10^4^ primary female or male HUVECs at a 1:1 cell-to-cell ratio for 48 and 72 hours in HUVEC complete medium. Additionally, in some approaches TGF-β was blocked by using a monoclonal mouse anti-human TGF-β 1,2,3 antibody (10 ng/μl; R&D Systems, Wiesbaden, Germany) with proven neutralizing activity^38^.

In all experiments, CD4^+^CD25^−^Foxp3^−^ T cells that were cultured alone in the respective cell number served as controls. Conversion into CD4^+^Foxp3^+^ Treg cells was evaluated by extra- and intracellular staining for CD4 and Foxp3 followed by flow cytometry analysis.

### Migration assay

Using a two-chamber trans-well system 5 × 10^4^ CD4^+^CD25^−^Foxp3^−^ T cells (upper chamber) were separated from 5 × 10^4^ primary female or male HUVECs (lower chamber) by a semi-permeable membrane with a pore size of 8 μm. The pore size allows CD4^+^CD25^−^Foxp3^−^ T cells to actively migrate to HUVECs. After 4, 8, 24 and 48 hours the number of migrated T cells into the lower chamber was counted by flow cytometry. Spontaneous migration of CD4^+^CD25^−^Foxp3^−^ T cells was assessed by culturing the cells in the absence of HUVECs.

### Determination of IL-10 levels by Cytometric Bead Array and TGF-β levels by Enzyme-linked immunosorbent assay (ELISA) in HUVEC culture supernatants

Supernatants from 1 × 10^4^ primary female or male HUVECs were collected after 48 and 72 hours and analyzed for IL-10 (using the Human Th1/Th2 Cytokine Kit II; BD Biosciences, Heidelberg, Germany) and TGF-β (using the Human/Mouse TGF beta1 ELISA Ready-Set-Go; Affymetrix eBiosciences, Frankfurt, Germany) levels. All steps were performed according to the instructions of the manufacturer.

### Flow cytometry analysis

CD4^+^CD25^−^Foxp3^−^ T cells were stained for the extracellular markers CD4 and CD25 as well as for the intracellular marker Foxp3. Extra- and intracellular staining procedure was conducted using our established staining protocols^39^. The following antibodies were used: PE-labeled CD4 (dilution 1:10; clone: RPA-T4), APC-labeled CD25 (dilution 1:5; clone: M-A251) and AF488-labeled Foxp3 (dilution 1:5; clone: 259D/C7). All antibodies were purchased from BD Pharmingen, Heidelberg, Germany. T cells were analyzed on a FACSCalibur from BD Biosciences, Heidelberg, Germany.

### Data analysis and statistics

Proliferation assays and the first set of conversion assays were performed 16 times (peripheral blood samples from 16 different normal pregnant women) in duplicates. Migration assays and the second and third set of conversion assays were conducted 2–4 times (peripheral blood samples from 9 normal pregnant women) in duplicates. Data presentation and analysis were performed with GraphPad Prism 5.0 software (Statcon, Witzenhausen, Germany). Data are presented as means ± standard error of the mean (S.E.M.). For T cell proliferation and conversion into Treg cells, controls (CD4^+^Foxp3^−^ T cells cultured alone) were set as “1” and co-cultures with HUVECs were calculated as fold change compared to controls. Statistical differences between the groups were analyzed using Two-way-ANOVA followed by Bonferroni correction for multiple comparisons.

## Additional Information

**How to cite this article**: Oettel, A. *et al*. Human Umbilical Vein Endothelial Cells foster conversion of CD4^+^CD25^−^Foxp3^−^ T cells into CD4^+^Foxp3^+^ Regulatory T Cells via Transforming Growth Factor-β. *Sci. Rep*. **6**, 23278; doi: 10.1038/srep23278 (2016).

## Supplementary Material

Supplementary Information
