# Supplementary material for: Human Umbilical Vein Endothelial Cells foster conversion of CD4+CD25−Foxp3− T cells into CD4+Foxp3+ Regulatory T Cells via Transforming Growth Factor-β
Source: Sci Rep. 2016 Mar 18;6:23278. doi: 10.1038/srep23278 (PMC4796866; doi:10.1038/srep23278)

Human Umbilical Vein Endothelial Cells foster conversion of $CD4^{+}CD25^{-}$ $Foxp3^{-}$ T cells into $CD4^{+}Foxp3^{+}$ Regulatory T Cells via Transforming Growth Factor-β

Anika Oettel[1,#], Mario Lorenz[2,3#], Verena Stangl[2,3], Serban-Dan Costa[4], Ana Claudia Zenclussen[1], Anne Schumacher[1,*]

[1]Experimental Obstetrics and Gynecology, Medical Faculty, Otto-von-Guericke University, Gerhart-Hauptmann-Straße 35, 39108 Magdeburg, Germany

[2]Medical Clinic for Cardiology and Angiology, Campus Mitte, Charite – Universitätsmedizin Berlin, Germany, Charitéplatz 1, 10117 Berlin, Germany

[3]DZHK (German Center for Cardiovascular Research), partner site Berlin, Oudenarder Straße 16, 13347 Berlin, Germany

[4]University Women´s Clinic, Otto-von-Guericke University, Gerhart-Hauptmann-Straße 35, 39108 Magdeburg, Germany

Supplementary Figure Legends

Supplementary Figure S1. Female HUVECs induced Treg cells from $CD4^+$ T cells

$CD4^+CD25^-Foxp3^-$ T cells were co-cultured with primary female HUVECs at cell-to-cell ratios of 1:1; 2:1 and 10:1 (T cells:HUVECs). After 48 and 72 hours, the number of $CD4^+Foxp3^+$ T cells was analyzed. At a cell-to-cell ratio of 1:1 female HUVECs significantly induced Treg cell generation at both time points. Results are presented as percentages (c). Representative flow cytometry plots are displayed for 48 hours (a) and 72 hours (b). Samples from 16 normal pregnant women were analyzed in duplicates. Data are presented as means ± S.E.M.. Statistical analysis among groups was performed using Two-way-ANOVA followed by Bonferroni correction for multiple comparisons. *=$p<0.05$; **=$p<0.01$.

Supplementary Figure S2. Male HUVECs induced Treg cells from $CD4^+$ T cells

$CD4^+CD25^-Foxp3^-$ T cells were co-cultured with primary male HUVECs at cell-to-cell ratios of 1:1; 2:1 and 10:1 (T cells:HUVECs). After 48 and 72 hours, the number of $CD4^+Foxp3^+$ T cells was analyzed. Male HUVECs induced only slightly and non-significantly the generation of Treg cells. Results are presented as percentages (c). Representative flow cytometry plots are displayed for 48 hours (a) and 72 hours (b). Samples from 16 normal pregnant women were analyzed in duplicates. Data are presented as means ± S.E.M.. Statistical analysis among groups was performed using Two-way-ANOVA followed by Bonferroni correction for multiple comparisons.

Supplementary Figure S3. HUVECs did not induce migration of $CD4^+$ T cells

Using a two-chamber trans-well system $CD4^+CD25^-Foxp3^-$ T cells (upper chamber) were separated from female or male HUVECs (lower chamber). After 4, 8, 24 and 48 hours, the number of migrated T cells to the lower chamber was determined. Neither female (a) nor male

(b) HUVECs induced migration of T cells. T cells cultured alone (controls) revealed some spontaneous T cell migration. Samples from two normal pregnant women were analyzed in duplicates. Data are presented as means ± S.E.M.. Statistical analysis among groups was performed using Two-way-ANOVA followed by Bonferroni correction for multiple comparisons.

Supplementary Figure S1

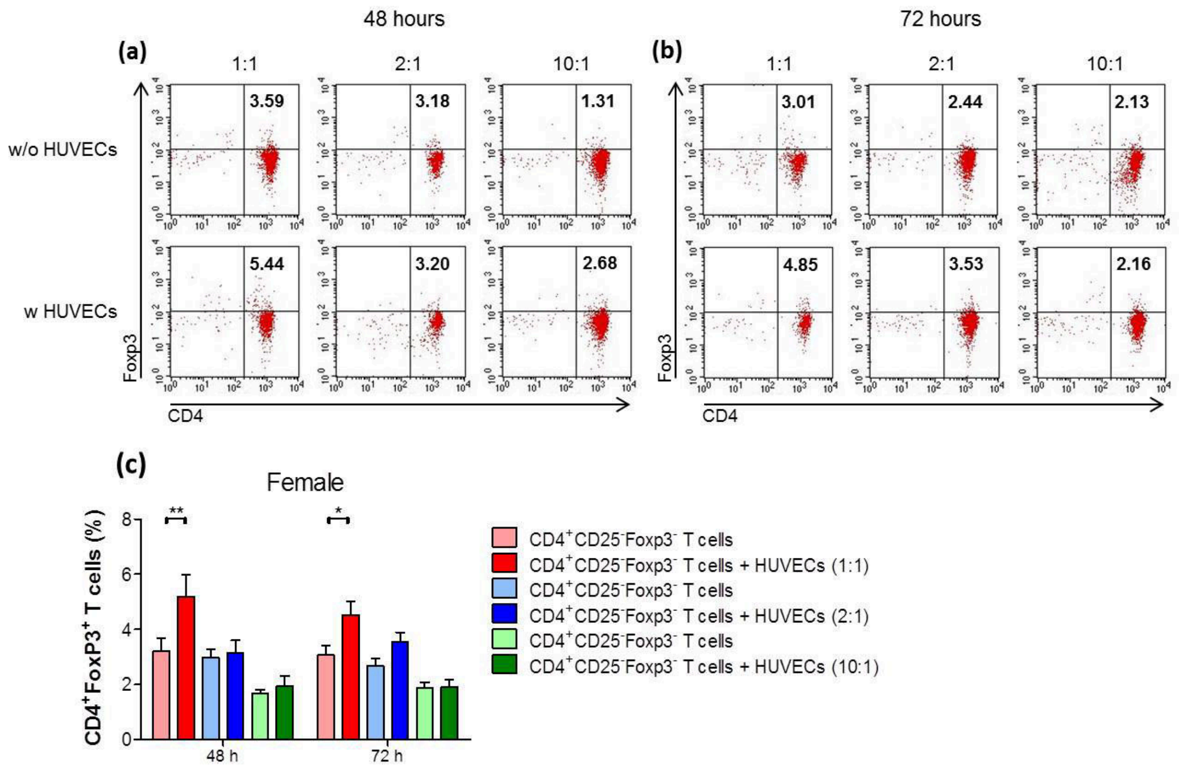

Supplementary Figure S2

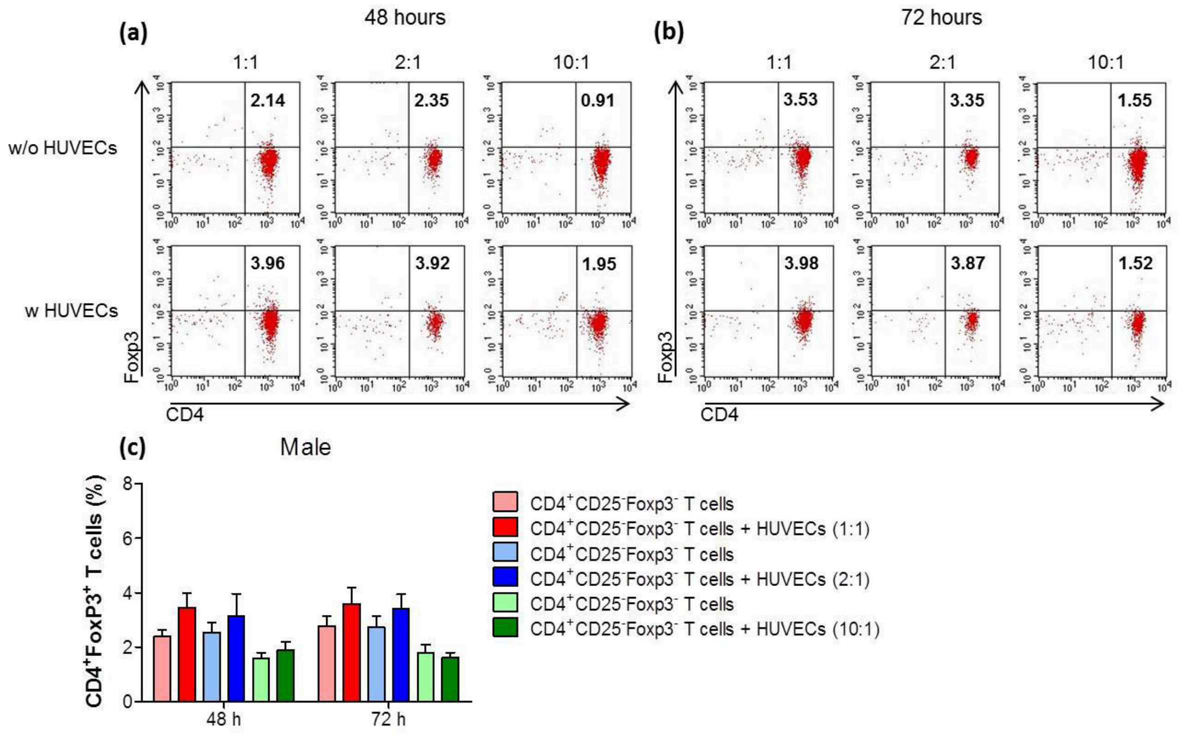

Supplementary Figure S3

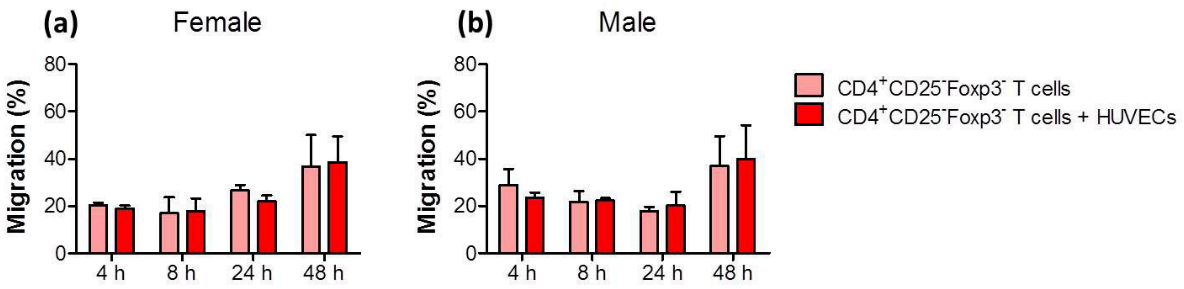

Supplement: Supplementary Information [file srep23278-s1.pdf]
